# Supplementary material for: Expression, activity, and consequences of biochemical inhibition of α- and β-glucosidases in different life stages of Culex quinquefasciatus
Source: PLoS One. 2023 Aug 29;18(8):e0286609. doi: 10.1371/journal.pone.0286609 (PMC10464971; doi:10.1371/journal.pone.0286609)
Supplement: Attachment — Submitted filename: Response to Reviewers.docx [file pone.0286609.s003.docx]

Response to reviewers:

We thank the reviewers for their helpful comments that have improved our manuscript. We have made comments below addressing the general review statement. Additional, Reviewer #2 sent along a document with grammatical comments. We have generated a Word document with Track Changes added to it documenting which changes we made and which we didn’t, along with an explanation for why we chose not to make the change. Comments below are preceded by ***.

**Reviewer #1: The results reported by this study are original and have not been published elsewhere and suggest the importance of α- and β-glucosidases on the survival in the 3 life stages of Cx. Quinquefasciatus studied. The experiments were described with sufficient details and the statistical analysis were well performed. The conclusions were supported accordingly to the data; however, the discussion may need a fresh literature for more clarity about the research in parallel that were done during the last few years. Therefore, I recommend a minor correction.**

***We thank this reviewer for the flattering words on our work. We were very unsure of what this reviewer means by “fresh literature” about the “research in parallel.” To our knowledge, and with extensive literature searches preceding the submission of this manuscript, we found very little recent work done on the topic of glucosidases in mosquitoes. Of the ones we found, we addressed them in the introduction. As it stands, we feel that the discussion addresses our discoveries based on what is known and accepted in the entomological literature on this topic. We will default to what the editor would like us to do, if anything, to address this comment.

**Reviewer #2: The manuscript is interesting and provides valuable insights into the role of α-glucosidases and β-glucosidases in the survival of Cx. quinquefasciatus mosquitoes in different life stages. The findings contribute to our understanding of mosquito digestion, species-specific knowledge, biocontrol agents, and potential applications for vector control strategies.
However, I suggested an English review, I added some comments in the pdf. file.**

***This reviewer provided a comprehensive review of our document’s grammar and syntax. We thank them for catching some errors and in general striving to improve the readability and flow of our manuscript. We have made a good number of the requested changes, while leaving others unchanged. We have added our changes and response to the comments in a Track Changes version of our manuscript.
